# Comparative study of the effects of the three kinds of Kampo-hozai: Ninjinyoeito, Hochuekkito, and Juzentaihoto on anxious and low sociability behavior using NPY-knockout zebrafish

**DOI:** 10.3389/fphar.2023.1168229

**Published:** 2023-05-30

**Authors:** Momoko Kawabe, Takumi Nishida, Ryuji Takahashi, Akio Inui, Kazuhiro Shiozaki

**Affiliations:** ^1^ Course of Biological Science and Technology, The United Graduate School of Agricultural Sciences, Kagoshima University, Kagoshima, Japan; ^2^ Pharmacological Department of Herbal Medicine, Graduate School of Medical and Dental Sciences, Kagoshima University, Kagoshima, Japan; ^3^ Department of Food Life Sciences, Faculty of Fisheries, Kagoshima University, Kagoshima, Japan; ^4^ Kampo Research Laboratories, Kracie Pharma Ltd., Toyama, Japan

**Keywords:** Kampo (traditional Japanese herbal medicine), zebrafish, anxious behavior, sociability, Ninjinyoeito, Hochuekkito, Juzentaihoto

## Abstract

Ninjinyoeito, Hochuekkito, and Juzentaihoto are the three types of Kampo-hozai used to support the treatment of various diseases by energizing patients through improved mental health. While Kampo-hozais are clinically used to improve mental energy decline, a comparison between their effects on neuropsychiatric symptoms like anxiety and sociability and the strength of their effects has not been conducted. Therefore, this study compared the effects of Ninjinyoeito, Hochuekkito, and Juzentaihoto on psychiatric symptoms using neuropeptide Y knockout (NPY-KO) zebrafish, a suitable animal model for anxiety and low sociability. Neuropeptide Y knockout zebrafish were fed a Ninjinyoeito, Hochuekkito, or Juzentaihoto-supplemented diet for 4 days. Then, sociability was analyzed using a three-Chambers test and anxiety-like behavior was evaluated using the cold stress and novel tank tests. The results showed that Ninjinyoeito treatment improved the low sociability of neuropeptide Y knockout, while Hochuekkito and Juzentaihoto did not. Neuropeptide Y knockout exhibited anxiety-like behaviors, such as freezing and swimming in the wall area under cold stress, but Ninjinyoeito treatment improved these behaviors. However, these anxiety-like behaviors were not improved by Hochuekkito and Juzentaihoto. Ninjinyoeito treatment also improved anxiety-like behaviors of neuropeptide Y knockout in the novel tank test. However, no improvement was shown in the Hochuekkito and Juzentaihoto groups. This trend was also confirmed in the low water stress test using wild-type zebrafish. This study exhibits that among the three types of Kampo-hozai, Ninjinyoeito is the most effective in psychiatric disorders associated with anxiety and low sociability.

## 1 Introduction

Kampo is a traditional Japanese herbal medicine that originates from Chinese medicine. Herbs that constitute Kampo are composed of multiple plants and sometimes minerals that contain many compounds. Kampo has attracted attention as an important therapeutic agent in clinical practice (Kiyomi et al., 2021). It can be used to treat a variety of conditions ranging from mental illnesses to physical frailty (Ushio et al., 2022). In fact, 70%–90% of Japanese physicians regularly prescribe Kampo (Kiyomi et al., 2021). It has multiple uses and is expected to have combined pharmacological effects (Zhou et al., 2016).

When physical and mental energies decline due to aging or illness, Kampo attempts to restore the body by compensating for the deficiency (Kiyomi et al., 2021). Kampo-hozai is a group of herbal medicines with complementary effects (Amitani, 2015). It supports the treatment of various diseases by enhancing patients’ mental health (Amitani, 2015; Kiyomi et al., 2021). It is characterized by the inclusion of Ginseng and Astragali Radix (Aomatsu et al., 2021; Miyazaki et al., 2021).

The most commonly used Kampo-hozai medicines in clinical practice are Ninjinyoeito (NYT), Hochuekkito (HET), and Juzentaihoto (JTT) (Amitani, 2015; Kiyomi et al., 2021). NYT comprises 12 herbal medicines, whereas HET and JTT comprise 10 herbal medicines (Table 1). These three Kampo-hozais have similar herb compositions: five common herbs (Ginseng, Japanese Angelica root, Atractylodes rhizome, Astragalus root, and Glycyrrhiza) (Kiyomi et al., 2021). The composition of NYT is similar to that of JTT, with nine common herbal medicines: Peony root, Rehmannia root, Poria sclerotium, and Cinnamon bark. NYT has been shown to improve frailty, anorexia, anxiety, and sociability in clinical studies and experimental animals (Tohda and Mingmalairak, 2013; Kawabe et al., 2021). HET is commonly used to treat malaise, anorexia, night sweats, and fever (Ohsawa et al., 2021). It also improves depressive symptoms, anorexia (Tohda and Mingmalairak, 2013), and anxiety-like behavior in LPS-induced inflammation in mice (Ushio et al., 2022). JTT is used to prevent anemia, loss of appetite, dry mouth, and side effects of cancer treatment such as anorexia and fatigue (Chino et al., 2005; Ohsawa et al., 2021). Although these are similar in composition and symptoms, no reports have directly compared their effects. While they are clinically used to improve decline in mental energy, a comparison of their effects on neuropsychiatric symptoms like anxiety and sociability and the strength of their effects have not been conducted. Unlike NYT, there are no reports of improvement in anxiety for JTT and in sociability for HET and JTT, despite their similar herb compositions. It is important to clarify how the effects of these medicines differ in terms of their responses in patients in clinical settings.

**TABLE 1:** Formulas of Ninjinyoeito (NYT), Hochuekkito (HET), and Juzentaihoto (JTT).

Component herbs	Family name	Species name	Weight (g)^a^		
			NYT^a^	HET^b^	JTT^c^
Rehmanniae Radix (Rehmannia root)	Plantaginaceae	*Rehmannia glutinosa* (Gaertn.) DC.	4	—	3
Angelicae acutilobae Radix (Japanese Angelica root)	Apiaceae	*Angelica acutiloba* (Siebold and Zucc.) Kitag	4	3	3
Atractylodis Rhizoma (Atractylodes rhizome)	Compositae	*Atractylodes japonica* Koidz. ex. Kitam	4	4	3
Poria (Poria sclerotium)	Polyporaceae	*Wolfiporia cocos* Ryvarden et Gilbertson	4	—	3
Ginseng Radix (Ginseng)	Araliaceae	*Panax ginseng* C.A. Mey	3	4	3
Cinnamomi Cortex (Cinnamon bark)	Lauraceae	*Cinnamomum cassia* (L.) J. Presl	2.5	—	3
Polygalae Radix (Polygala root)	Polygalaceae	*Polygala tenuifolia* Wild	2	—	—
Paeoniae Radix (Peony root)	Paeoniaceae	*Paeonia lactiflora* Pall	2	—	3
Citri unshiu Pericarpium (Citrus unshiu peel)	Rutaceae	*Citrus unshiu* Markowicz	2	2	—
Astragali Radix (Astragalus root)	Polygalaceae	*Astragalus mongholicus* Bunge	1.5	4	3
Glycyrrhizae Radix (Glycyrrhiza)	Fabaceae	*Glycyrrhiza uralensis* Fisch. ex DC.	1	1.5	1.5
Schisandrae Fructus (Schisandra fruit)	Schisandraceae	*Schisandra chinensis* (Turcz.) Baill	1	—	—
Bupleuri Radix (Bupleurum Root)	Apiaceae	*Bupleurum falcatum* Linné	—	2	—
Cimicifuga rhizome (Cimicifuga rhizome)	Ranunculaceae	*Cimicifuga simplex* Turczaninow	—	1	—
Ziziphi Fructus (Jujube)	Rhamnaceae	*Ziziphus jujuba* Miller var. *inermis* Rehder	—	2	—
Zingiberis Rhizoma (Ginger)	Zingiberaceae	*Zingiber officinale* Roscoe	—	0.5	—
Cnidii Rhizoma (Cnidium rhizome)	Umbelliferae	*Cnidium officinale* Makino	—	—	3

^a^ Amount of herbs for the preparation of 6.7 g NYT extract, daily dosage for human.

^b^ Amount of herbs for the preparation of 6.4 g HET extract, daily dosage for human.

^c^ Amount of herbs for the preparation of 6.2 g JTT extract, daily dosage for human.

Recently, zebrafish have attracted attention and are widely used in behavioral neuroscience research (Rosa et al., 2018). They have many advantages such as ease of maintenance, low cost, and abundant offspring (Rosa et al., 2018). They exhibit anxiety-like behavior and high sociability similar to rodents (Rosa et al., 2018; Ariyasiri et al., 2019). In addition, they possess many genes involved in the regulation of anxiety and sociability in humans and rodents (Rosa et al., 2018). They exhibit anxiety-like behaviors, such as freezing under acute stress, and can be evaluated similar to rodents. They have established protocols for assessing sociability using the three-Chambers test (Ariyasiri et al., 2019). Thus, they are suitable for drug discovery research and used in Kampo medicine research (Kawabe et al., 2021; Kawabe et al., 2022). Recently, neuropeptide Y-knockout (NPY-KO) zebrafish were established as models for anxiety-like and low social behavior (Shiozaki et al., 2020). NPY is involved in the regulation of emotional behaviors such as social and anxiety behaviors in a mammal (Ueda et al., 2021). The zebrafish NPY amino acid sequence and the NPY function are very similar to the human NPY (Singh et al., 2017; Shiozaki et al., 2020). The NPY-KO zebrafish showed low sociability in the three-Chambers test and exhibited anxiety-like behaviors such as freezing under acute stress (Kawabe et al., 2022).

This study compares the effects of NYT, HET, and JTT on psychiatric symptoms, such as anxiety and sociability, using NPY-KO zebrafish, a suitable animal model for anxiety and low sociability. Not only is the use of different Kampo-hozai for neuropsychiatric symptoms empirical but their effects have not yet been compared.

## 2 Materials and methods

### 2.1 Zebrafish

The NPY-KO zebrafish were generated by genome editing using the RIKEN WT (RW) strain and were accompanied by the deletion of 11 nucleotides in *npy* in previous reports (Shiozaki et al., 2020). The RW strain was used as the WT strain in this study. The zebrafish were reared in a 2-L water tank with a 14/10 h light/dark photoperiod cycle, and live brain shrimp and a commercial diet (Otohime B2, Marubeni Nissin Feed Ltd., Tokyo, Japan) were provided twice daily. Adult (6–12 months old) zebrafish were used. All animal experiments were approved by the Kagoshima University Committee (ethics protocol No. F19002), and this study was conducted following the relevant guidelines and regulations.

### 2.2 Administration of NYT, HET, and JTT in zebrafish

NYT (Lot No. 16033006), HET (Lot No. E2102051A0), and JTT (Lot No. KS-540) were provided as freeze-dried powders in boiling water extract by Kampo Research Laboratories (Kracie Pharma, Ltd., Toyama, Japan). All herbs that comprise the three Kampo-hozais were listed in Table 1. Each plant material was identified by external morphology and authenticated by marker compounds of plant specimens according to the method of Japanese Pharmacopeia and our company’s standard. Quality check of NYT and the extraction ratio were described in our previous study (Kawabe et al., 2022). For the quality check of HET and JTT, the extracts were mixed and shaken with 50% methanol and the filtrates were subjected to high performance liquid chromatography (HPLC) analysis. The three-dimensional HPLC profiles of HET and JTT were obtained using a Shimazu Nexera X2 system with an SPD-M30A detector with scanning for a range of 190–450 nm and a reversed-phase column [ACQUITY UPLC^®^ BEH C18 1.7 μm (2.1 mm × 100 mm, 1.7 μm), Column temperature: 40°C]. The column was equipped with solvent A (0.1% formic acid in water) and solvent B (0.1% formic acid in methanol), and the ratio of solvent A was increased by A/B 90/10-90/10-5/95 (0–10–45 min), with a flow rate at 0.3 ml/min (Kawabe et al., 2022). The extraction ratio was 26.7% and 21.8% in HET and JTT, respectively.

Each Kampo-hozai medicine was mixed with the commercial diet at 3% or 0.3%. The used concentrations were decided according to previous studies using mice and zebrafish (Amitani et al., 2022; Kawabe et al., 2022). When the 3% supplemented diet is administered to zebrafish, the daily intake of Kampo-hozai is approximately equal to about 10 times the equivalent of the clinical use in humans. Control diets were prepared as above without NYT, HET, or JTT. This study did not provide a positive control group because drugs that specifically improve social skills or acute stress are poorly defined. The diets were stored at −20°C during the feeding experiments.

Feeding experiments were conducted in 2-L tanks filled with water at 28°C. Zebrafish (average body weight 0.7 g) were fed a commercial diet until the start of the feeding experiment and then divided into 2-L tanks. The experimental diets were fed twice daily for 4 days. The food intake of the zebrafish was recorded daily.

### 2.3 Locomotor performance

Behavioral tests were conducted to examine the effects of NYT, JTT, and HET administration on locomotor performance. Wild zebrafish were acclimated in a test tank (6 cm high, 20 cm wide, and 18.5 cm long) for 15 min, and the total distance traveled for motility was analyzed for 3 min. The NPY-KO zebrafish were acclimated in a test tank (7 cm high, 18 cm wide, and 23.5 cm long) for 15 min, and the total distance traveled for motility was analyzed for 3 min.

### 2.4 Three-chambers test

The sociability of Kampo-hozai-fed NPY-KO zebrafish was analyzed using a three-Chambers test (Ikeda et al., 2021). The experimental scheme used for the three-Chambers test is shown in Figures 2A, B. On one side of the test tank (7 cm high, 18 cm wide, and 23.5 cm long), two small transparent chambers (5.5 cm high, 9.1 cm wide, and 5.3 cm long) were placed, called the fish and empty chamber, respectively. The fish chamber was filled with two male and two female zebrafish and the empty chamber was set as empty. A test fish was placed in the center of the tank, transferred to a test tank with the front of the chamber covered with a white board, and acclimated for 10 min. The white board was then removed, and the behavior of the fish was recorded for 5 min with a digital video camera. Move-tr/2D software (Library, Tokyo, Japan) recorded all the dates of the time spent in the fish and empty chamber area (0.5 cm area in front of the fish and empty chamber area), the total distance traveled, and swimming tracking.

### 2.5 Cold stress test

Anxiety in Kampo-hozai-fed NPY-KO zebrafish was analyzed using a cold stress test. It was used to analyze the anxiety-like behavior under acute stress of NPY-KO zebrafish (Shiozaki et al., 2020; Kawabe et al., 2021). The experimental scheme used for the test is shown in Figure 3A. The fish were placed in water at 10°C for 2 s, and then transferred to the test tank (5 cm high, 10 cm wide, and 24 cm long) made of white opaque plastic. Freezing time and swimming tracking were analyzed for 10 min, and the total distance traveled for 3 min immediately after the transfer to the test tank. Subsequently, the total distance and time spent in the wall area (2 cm within the wall) were analyzed for 2 min after transfer to the test tank and acclimation for 15 min. The fish’s behavior was analyzed using Move-tr/2D software.

### 2.6 Novel tank test

The anxiety in Kampo-hozai-fed NPY-KO zebrafish was analyzed using a novel tank test (Rosa et al., 2018). The experimental scheme used for the test is shown in Figure 4A. After the fish were transferred to the test tank (17.5 cm high, 28 cm wide, and 14 cm long), freezing time and swimming tracking were analyzed for 10 min, and the total distance traveled for 3 min. After observing freezing behavior for 10 min, the total distance, time spent in the upper area, and total number of entries into the upper area were analyzed for 3 min. The fish’s behavior was analyzed using Move-tr/2D software.

### 2.7 Low water level stress test

A low-water level stress test was used to analyze anxiety-like behaviors in wild zebrafish (Yan et al., 2016). The experimental scheme used for the test is shown in Figure 5A. The fish were allowed to swim for 2 min at the water level so that their dorsal surface was exposed to the water surface (about 0.7 cm depth) and then transferred to the test tank (6 cm high, 20 cm wide, and 18.5 cm long). Freezing time and swimming tracking were analyzed for 10 min, and the total distance traveled for 3 min after transfer to the test tank. Subsequently, the total distance and time spent in the wall area (2 cm within the wall) were analyzed for 2 min after habituation in the test tank for 15 min. The fish’s behavior was analyzed using Move-tr/2D software.

### 2.8 Data analysis

Results are demonstrated as mean ± standard deviation. All values were compared using a one-way analysis of variance (ANOVA) followed by Tukey’s multiple comparison test.

## 3 Results

### 3.1 Comparison of the effects of the three Kampo-hozais on low sociability in NPY-KO zebrafish

Supplementary Figures 1, 2 show 3D-HPLC profiles of HET and JTT along with a chemical analysis. Chemical makers, such as paeoniflorin, hesperidin, and glycyrrhizic acid, were used for quality control. Comparison with standards using DAD spectra confirmed the presence of reference compounds in HET and JTT.

**FIGURE 1:**
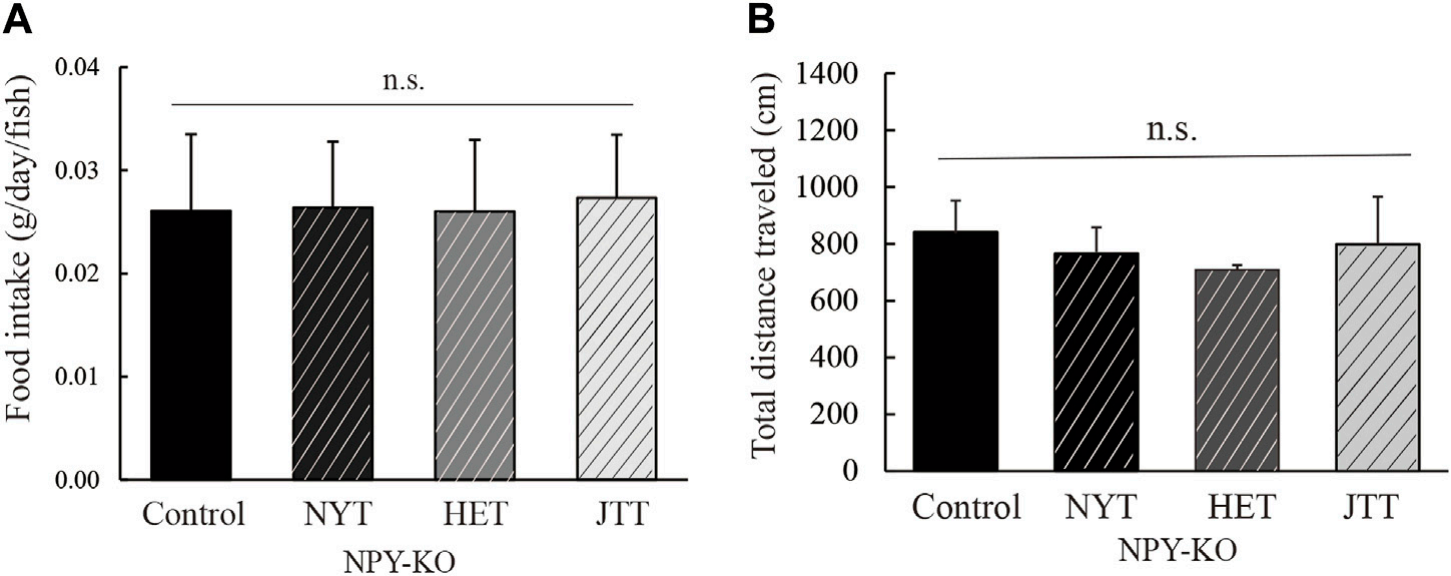
Effect of the three Kampo-hozai on the appetite and mobility in NPY-KO zebrafish. **(A,B)** The NPY-KO zebrafish were fed control, NYT, HET, or JTT twice daily for 4 days (3% concentration of NYT, HET, or JTT in the diet). Controls were fish fed feed without Kampo-hozai. **(A)** Food intake, *n* = 8. **(B)** Swimming mobility was estimated using the total distance traveled, *n* = 4. Results are shown as mean ± standard deviation. n.s., not significant.

NYT-, HET-, and JTT-fed zebrafish were examined for their effects on food intake and mobility under non-stress conditions because Kampo-hozais has been prescribed for patients with post-illness or post-operative weakness, fatigue, and loss of appetite. Food intake and total distance traveled were not altered by the feeding of three Kampo-hozais compared to the control diet (Figures 1A, B), indicating that using Kampo-hozais did not affect their appetite and mobility in zebrafish.

**FIGURE 2:**
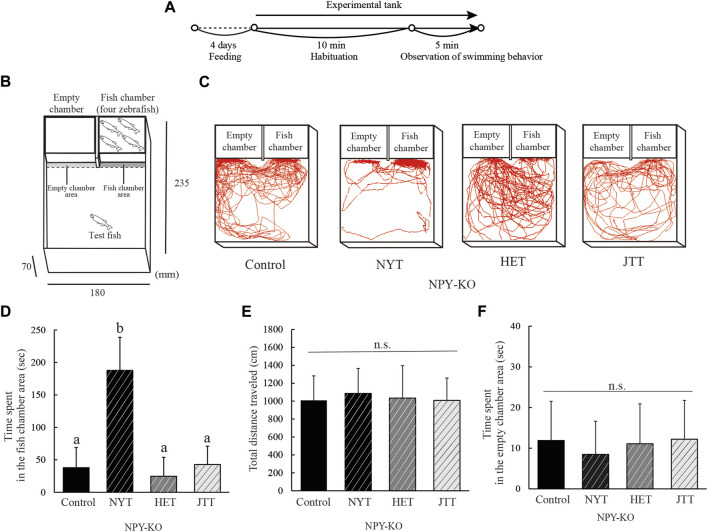
Comparison of the effects of the three Kampo-hozai on low sociability in NPY-KO zebrafish. **(A–F)** The NPY-KO zebrafish were fed control, NYT, HET, or JTT twice daily for 4 days (3% concentration of NYT, HET, or JTT in the diet). Controls were fish fed feed without Kampo-hozai. Sociability was assessed using a three-Chambers test. **(A)** Experimental scheme for behavioral evaluation. **(B)** Test apparatus. **(C)** Tracking of swimming behavior of fish fed control, NYT, HET, and JTT. **(D)** Time spent in the fish chamber area by the control and three Kampo-hozai-fed NPY-KO zebrafish. **(E)** Total distance traveled by the control and three Kampo-hozai-fed NPY-KO zebrafish. **(F)** Time spent in the empty chamber area by the control and three Kampo-hozai-fed NPY-KO zebrafish. Control *n* = 11, NYT *n* = 9, HET *n* = 6, JTT *n* = 6. Results are shown as mean ± standard deviation. n.s., not significant. (a,b) Groups with different letters in the same row are significantly different by one-way ANOVA-test with Tukey’s multiple comparison test (*p* < 0.01).

To compare the effects of NYT, HET, and JTT on sociability, we conducted a three-Chamber test using NPY-KO zebrafish that exhibited low sociability (Kawabe et al., 2022). As shown in Figure 2C, NYT-fed zebrafish showed an increase in the time spent in the fish chamber area compared to the control diet (6.2-fold increase; F = 39.348, *p* < 0.0001, one-way ANOVA; *p* < 0.01), whereas HET- and JTT-fed zebrafish did not show any alteration in their interaction with the fish chamber (Figure 2D). None of the tested Kampo-hozai extracts affected the total distance traveled (Figure 2E) or the time spent in the empty chamber in NPY-KO zebrafish compared to the control (Figure 2F). These results suggest that among the three Kampo-hozai, only NYT exhibited an improvement in sociability.

### 3.2 Comparison of anxiolytic effects of the three Kampo-hozais

#### 3.2.1 Comparison of the effects of the three Kampo-hozais on anxiety-like behavior using the cold stress test in NPY-KO zebrafish

**FIGURE 3:**
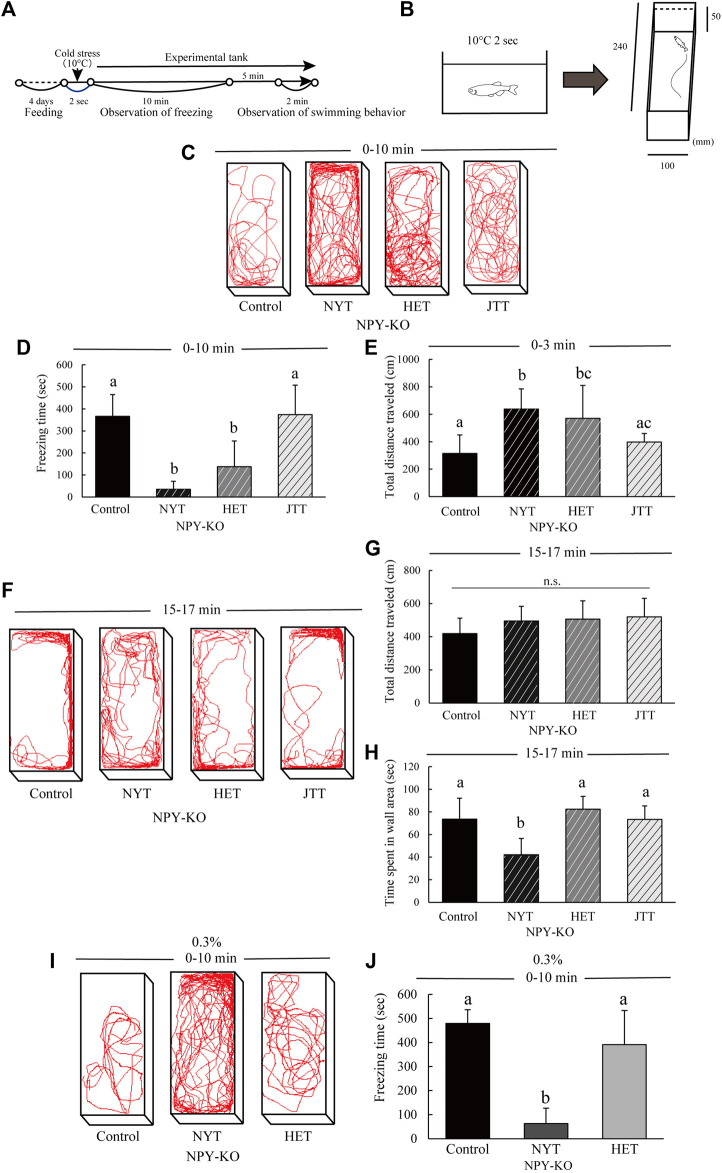
Comparison of the effects of the three Kampo-hozai on anxiety-like behavior using cold stress test in NPY-KO zebrafish. **(A–J)** The NPY-KO zebrafish were fed control, NYT, HET, or JTT twice daily for 4 days (3% concentration of NYT, HET, and JTT in diet). Controls were fish fed feed without Kampo-hozai. Anxiety-like behaviors, such as freezing and swimming in the wall area, were estimated after the cold stress test. **(A)** Experiment scheme for behavioral evaluation. **(B)** Test method and apparatus. **(C)** Tracking of control, NYT, HET, and JTT swimming behavior in 0–10 min (3% concentration of NYT, HET, JTT in diet). **(D)** Total freezing time by control and the three Kampo-hozai-fed NPY-KO zebrafish. **(E)** Total distance traveled by control and the three Kampo-hozai-fed NPY-KO zebrafish in 0–3 min. Control *n* = 10, NYT *n* = 10, HET *n* = 5, JTT *n* = 5. **(F)** Tracking of control, NYT, HET, and JTT swimming behavior in 15–17 min. **(G)** Total distance traveled by control and the three Kampo-hozai-fed NPY-KO zebrafish in 15–17 min. **(H)** Time spent in the wall area of the control and three Kampo-hozai-fed NPY-KO zebrafish. **(I)** Tracking of control, NYT, HET, and JTT swimming behavior in 0–10 min (0.3% concentration of NYT or HET in diet). **(J)** Total freezing time by control and the three Kampo-hozai-fed NPY-KO zebrafish. Control *n* = 9, NYT *n* = 8, HET *n* = 9. Results are shown as mean ± standard deviation. n.s., not significant. (a,b,c) Groups with different letters in the same row are significantly different by one-way ANOVA-test with Tukey’s multiple comparison test [*p* < 0.05 in **(E)**, and *p* < 0.01 in **(D,H,J)**].

To compare the anxiolytic effects of NYT, HET, and JTT, they were fed to NPY-KO zebrafish and their behavior under stress was evaluated. Under acute stress, the NPY-KO zebrafish exhibit anxiety-like behaviors including freezing, increased swimming in the wall area of the test tank, and erratic movement (Shiozaki et al., 2020). In general, these behaviors are also observed in anxious mice as well. Alterations in anxiety-like behavior induced by cold stress were analyzed in Kampo-hozai-fed NPY-KO zebrafish (Figures 3A, B). After exposure to cold water, NYT- and HET-fed NPY-KO zebrafish decreased freezing time compared to the control (90.4% and 62.5%, respectively; F = 28.143, *p* < 0.0001, one-way ANOVA; *p* < 0.01, Figures 3C, D). However, JTT-fed NPY-KO zebrafish did not show any difference compared to the control group (Figures 3C, D). NYT- and HET-fed zebrafish showed an increase in the total distance traveled compared to the control (2- and 1.8-fold increase, respectively; F = 8.521, *p* = 0.000415, one-way ANOVA; *p* < 0.05, Figure 3E). These results indicate that NYT- and HET-treated zebrafish showed an increase in the total distance traveled owing to suppression of freezing. Next, we analyzed swimming behavior after the recovery from freezing. While HET- and JTT-fed zebrafish did not differ in the total distance traveled (Figures 3F, G), NYT-fed zebrafish exhibited a decrease in time spent in the wall area compared to the control (42.7% decrease; *p* < 0.05; F = 9.047, *p* = 0.000548, one-way ANOVA; *p* < 0.01, Figures 3F–H). HET- and JTT-fed zebrafish did not differ in time spent in the wall area from control-fed zebrafish (Figures 3F–H).

To determine whether NYT or HET resulted in more anxiolytic Kampo-hozai, we evaluated the anxiety-like behavior of NPY-KO zebrafish fed with a diet containing 0.3% NYT or HET. The NYT treatment decreased the freezing time compared to the control diet (86.9% decrease; F = 42.751, *p* < 0.0001, one-way ANOVA; *p* < 0.01, Figures 3I, J). The HET did not differ in freezing time from the control diet (Figures 3I, J). These results suggest that NYT has a stronger anxiolytic effect than HET or JTT in NPY-KO zebrafish subjected to acute cold stress.

#### 3.2.2 Comparison of the effects of the three Kampo-hozais on anxiety-like behavior using novel tank test in NPY-KO zebrafish

**FIGURE 4:**
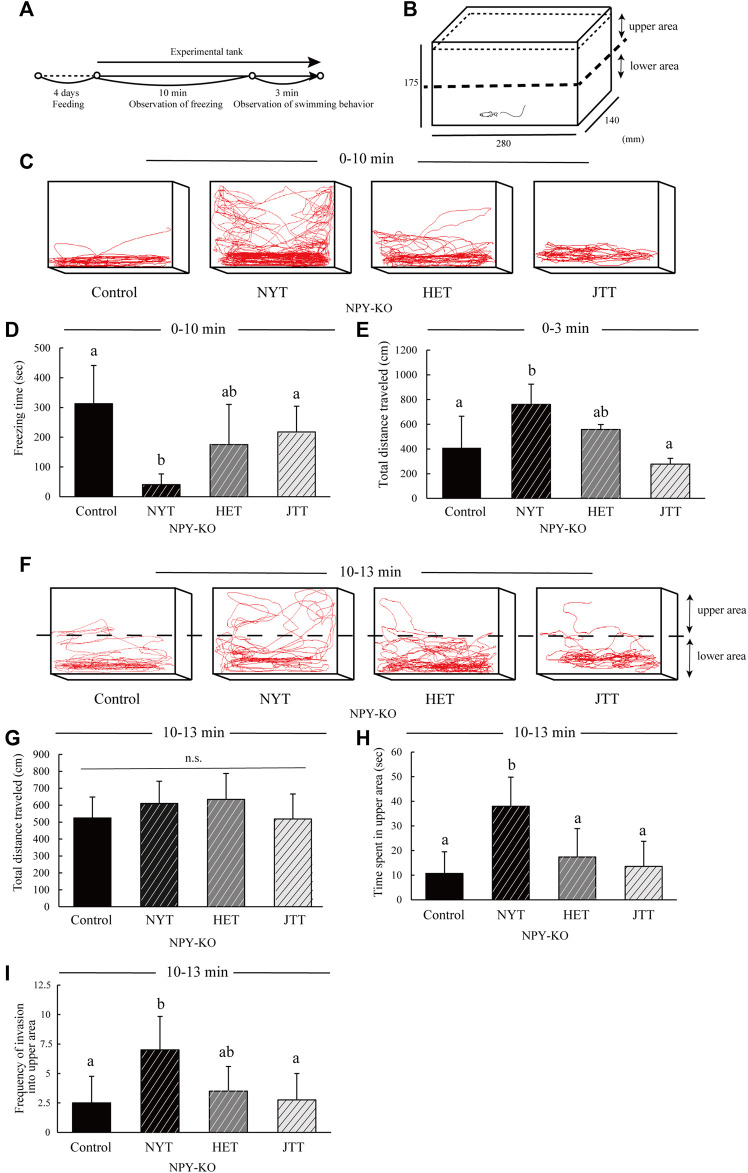
Comparison of the effects of the three Kampo-hozai on anxiety-like behavior using novel tank test in NPY-KO zebrafish. **(A–I)** The NPY-KO zebrafish were fed control, NYT, HET, or JTT twice daily for 4 days (3% concentration of NYT, HET, or JTT in the diet). Controls were fish fed feed without Kampo-hozai. Anxiety-like behaviors, such as freezing and swimming in the upper area, were assessed after the novel environmental stress. **(A)** Experimental scheme for behavioral evaluation. **(B)** Test apparatus. **(C)** Tracking of control, NYT, HET, and JTT swimming behavior at 0–10 min (3% concentration of NYT, HET, and JTT in diet). **(D)** Total freezing time by control and the three Kampo-hozai-fed NPY-KO zebrafish. **(E)** Total distance traveled by the control and three Kampo-hozai-fed NPY-KO zebrafish at 0–3 min *n* = 8. **(F)** Tracking of control (left), NYT (middle), HET (middle), and JTT (right) swimming behavior at 10–13 min. **(G)** Total distance traveled by the control and three Kampo-hozai-fed NPY-KO zebrafish. **(H)** Time spent in the upper area by the control and three Kampo-hozai-fed NPY-KO zebrafish. **(I)** Frequency of invasion into the upper area in the control and three Kampo-hozai-fed NPY-KO zebrafish. *n* = 7. Results are shown as mean ± standard deviation. n.s., not significant. (a,b) Groups with different letters in the same row are significantly different by one-way ANOVA-test with Tukey’s multiple comparison test (*p* < 0.05).

To evaluate the anxiolytic effects of the three Kampo-hozais on other stresses, a novel tank test was conducted using NPY-KO zebrafish (Figures 4A, B). NYT treatment showed a decreased freezing time compared to the control diet (87.0% decrease; F = 9.164, *p* = 0.000201, one-way ANOVA; *p* < 0.05, Figures 4C, D), whereas HET- and JTT-fed zebrafish did not differ from the control diet in freezing time (Figures 4C, D). NYT-fed zebrafish showed an increase in total distance traveled compared to the control (1.9-fold increase; F = 6.404, *p* = 0.006721, one-way ANOVA; *p* < 0.05, Figure 4E) because of reduced freezing behavior.

After the fish recovered from freezing behavior (approximately 10 min), we evaluated the total distance traveled, time spent, and total entries and frequency of invasion into the upper area (Figures 4F–I). The total distance traveled did not differ between NYT-, HET-, and JTT-treated zebrafish and the control (Figure 4G). NYT-fed zebrafish exhibited an increase in the time spent (3.6-fold increase, respectively; F = 8.016, *p* = 0.000948, one-way ANOVA; *p* < 0.05, Figure 4H) and frequency of invasion in the upper area compared to the control diet (2.8-fold increase; F = 4.603, *p* = 0.013181, one-way ANOVA; *p* < 0.05, Figure 4I). HET-and JTT-treated zebrafish did not differ in the time spent and frequency of invasion in the upper area compared to the control (Figures 4H, I). These results suggest that among the three Kampo-hozais, NYT exhibits anxiolytic effects in the NPY-KO zebrafish subjected to a novel environment.

#### 3.2.3 Comparison of the effects of the three Kampo-hozais on anxiety-like behavior using low water level stress test in wild zebrafish

**FIGURE 5:**
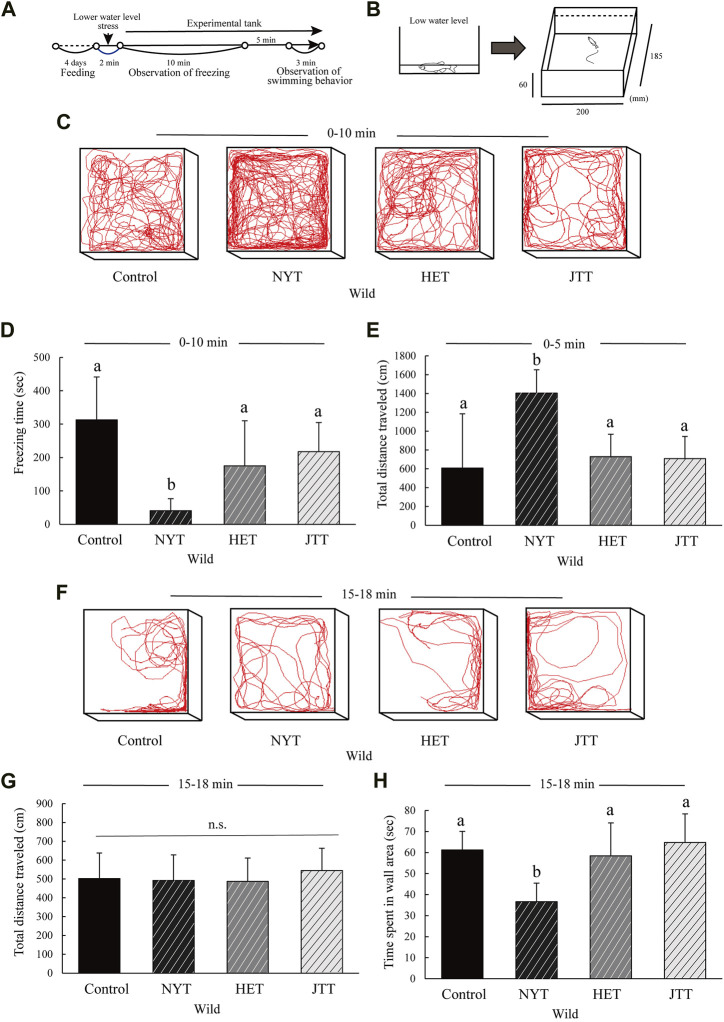
Comparison of the effects of the three Kampo-hozai on anxiety-like behavior using low water level stress test in wild zebrafish. **(A–H)** Wild zebrafish were fed control, NYT, HET, or JTT twice daily for 4 days (3% concentration of NYT, HET, or JTT in the diet). Controls were fish fed feed without Kampo-hozai. Anxiety-like behaviors, such as freezing and swimming in the wall area, were estimated after low water level stress. **(A)** Experimental scheme for behavioral evaluation. **(B)** Test methods and equipment **(C)** Tracking of control, NYT, HET), and JTT swimming behavior at 0–10 min (3% concentration of NYT, HET, and JTT in diet). **(D)** Total freezing time in the control and three Kampo-hozai-fed wild zebrafish. *n* = 8. **(E)** Total distance traveled by the control and three Kampo-hozai-fed wild zebrafish at 0–5 min. *n* = 9. **(F)** Tracking of control, NYT, HET, and JTT swimming behavior at 15–18 min. **(G)** Total distance traveled by the control and three Kampo-hozai-fed wild zebrafish. **(H)** Time spent in the wall area by the control and three Kampo-hozai-fed wild zebrafish. *n* = 6. Results are shown as mean ± standard deviation. n.s., not significant. (a,b) Groups with different letters in the same row are significantly different by one-way ANOVA-test with Tukey’s multiple comparison test [*p* < 0.05 in **(H)**, and *p* < 0.01 in **(D,E)**].

This study revealed the anxiolytic activity of NYT in NPY-KO zebrafish. NYT is reported to be involved in the activation of NPY neurons. To clarify whether the action of Kampo-hozai was not a phenomenon observed only in NPY-KO, we evaluated the anxiolytic effect of NYT using wild-type zebrafish with normal NPY using the low water stress test (Figures 5A, B) because, in our previous and preliminary experiments, wild zebrafish did not exhibit anxiety-like behavior in the cold stress and the novel tank test (Kawabe et al., 2021). The three Kampo-hozais did not affect food intake or total distance traveled in wild zebrafish, similar to the control diet (Supplementary Figures S3A, B). When wild zebrafish were exposed to low water stress (Figures 5A, B), NYT treatment decreased freezing time compared to the control (69.4% decrease; F = 14.574, *p* < 0.0001, one-way ANOVA; *p* < 0.01, Figures 5C, D). However, HET- and JTT-fed zebrafish did not differ in freezing time compared to the control (Figures 5C, D). Additionally, NYT-fed zebrafish showed an increase in the total distance traveled compared to the control (2.0-fold increase; F = 6.635, *p* = 0.001246, one-way ANOVA; *p* < 0.01, Figure 5E), possibly due to a decrease in freezing. After the fish recovered from freezing behavior, swimming behavior was analyzed in NYT-, HET-, and JTT-fed zebrafish (Figures 5F–H). The total distance traveled did not differ between the NYT, HET, and JTT treatments and the control diet (Figure 5G). The NYT-fed zebrafish exhibited a decrease in time spent in the wall area compared to the control (40.3% decrease; F = 7.312, *p* = 0.001411, one-way ANOVA; *p* < 0.05, Figures 5F–H). However, HET- and JTT-fed zebrafish did not differ in time spent in the wall area from the control (Figures 5F, H). These results suggest that among the three Kampo-hozais, NYT exhibits the most anxiolytic effect not only in NPY-KO zebrafish but also in wild zebrafish.

## 4 Discussion

NYT, HET, and JTT are similar in composition to other herbal medicines (Kiyomi et al., 2021). In this study, from clinical and basic research perspectives, we compared their effects on anxiety and sociability in NPY-KO zebrafish. As a result, NYT exhibited improved anxiety and sociability, different from HET and JTT.

Previous reports have shown that several herbs contained in NYT, such as Ginseng and Citrus Unshu Peel exhibit an anxiolytic effect (Lee and Rhee, 2017; Kawabe et al., 2021). Ginseng is a common herb in NYT, HET, and JTT, and Citrus Unshu Peel is in NYT and HET. However, the present study revealed that NYT showed more potent anxiolytic activity than HET and JTT. By the comparison of herb components in the three, Schisandra fruit is contained only in NYT. Our previous study revealed that Schisandra fruit exhibits the most potent anxiolytic activity among the herbs contained in NYT (Kawabe et al., 2021). On the other hand, Polygala root is also an herb unique to NYT but does not show any anxiolytic activity in zebrafish (Kawabe et al., 2021). Schisandra fruit has been widely used to treat neurological disorders such as insomnia and Alzheimer’s disease (Yan et al., 2016). It shows antidepressant-like effects via noradrenergic, dopaminergic, and GABAergic systems in mice (Yan et al., 2016). Our previous study has shown that schisandrin is one of the active components in Schisandra fruit (Kawabe et al., 2021). Schisandrin reduces the expression of tyrosine hydroxylase (*th1*), a marker of catecholamine neurons under acute stress (Kawabe et al., 2021). Under acute stress, the amount of catecholamines including noradrenalin elevates in zebrafish and mice brains (Silberman et al., 2003; Eto et al., 2014), and increased noradrenaline induces anxiety-like behavior, such as freezing (Browne et al., 2014). In addition, schisandrin rescues depression-like behaviors via the GDNF/ERK1/2/ROS and PI3K/AKT/NOX signaling pathways in mice (Wan et al., 2017). HET improves LPS-induced anxiety in mice, and the active herb is identified as glycyrrhiza (Ushio et al., 2022). The glycyrrhiza amount in HET was 1.5 times higher than in NYT (Kiyomi et al., 2021). Our previous study identified glycyrrhiza as an anxiolytic herb in NYT, but its activity was lower than the Schisandra fruit (Kawabe et al., 2021). Although NYT and JTT have many common herbal components, JTT has fewer anxiolytic effects than NYT. Thus, Schisandra fruit would be crucial as an active anxiety suppressant in the NYT, even though several herbal medicines that are known to suppress anxiety are included in the three herbal medicines. The purpose of this paper is to compare the effects of the three Kampo-hozai and to see if the effects can be explained by the differences in their constituent herbs. Therefore, a detailed mechanistic analysis was not performed in this paper.

HET and JTT have not reported any improvement in sociability, unlike NYT. This study found that NYT, but not HET and JTT, exhibited the effect of sociability improvement. Polygala root is a unique herb contained in NYT, not found in HET or JTT. We have previously identified that the polygala root in NYT is responsible for the improvement of sociability in NPY-KO zebrafish by regulating the hypothalamic-pituitary-adrenal axis (HPA) and/or sympathetic adrenal medullary axis (SAM) (Kawabe et al., 2022). Although Polygala root ameliorates anxiety and stress in mice by suppressing the HPA system and reducing noradrenaline (Lee et al., 2015), Schisandra fruit did not improve sociability in zebrafish (Kawabe et al., 2021). Because the active component(s) in Polygala root has not been identified, the molecular mechanism of NYT is unclear for the improvement of sociability. This study did not conduct the sociability tests with wild zebrafish because wild zebrafish originally exhibit high sociability (Kawabe et al., 2022) and is not suitable for the evaluation of Kampo-hozais.

In conclusion, this study provides evidence for the concomitant use of Kampo-hozai for a variety of disorders including psychiatric disorders associated with anxiety and low sociability. Among the three Kampo-hozais, NYT exhibited the most potent anxiolytic activity and recovered sociability. However, one limitation of the present study should be noted. Although molecules related to human drug metabolism are conserved in zebrafish (Bresolin et al., 2005; Machado et al., 2014), differences in digestion, absorption, and metabolism of drugs are not clearly defined between mammals and fish. Studies on the kinetics of drugs after absorption and identification of enzymes involved in drug metabolism have rarely been performed in zebrafish and require further investigation.

## Data Availability

The original contributions presented in the study are included in the article/Supplementary Material, further inquiries can be directed to the corresponding author.
