# Supplementary material for: Comparative study of the effects of the three kinds of Kampo-hozai: Ninjinyoeito, Hochuekkito, and Juzentaihoto on anxious and low sociability behavior using NPY-knockout zebrafish
Source: Front Pharmacol. 2023 May 30;14:1168229. doi: 10.3389/fphar.2023.1168229 (PMC10267730; doi:10.3389/fphar.2023.1168229)
Supplement: Supplementary file 1 [file DataSheet1.PDF]

Supporting information

Comparative study of the effects of the three kinds of Kampo-hozai: Ninjinyoeito, Hochuekkito, and Juzentaihoto on anxious and low sociability behavior using NPY-knockout zebrafish

Momoko Kawabe [a, d], Takumi Nishida [b], Ryuji Takahashi [c], Akio Inui [d] and Kazuhiro Shiozaki [a,b]

a Course of Biological Science and Technology, The United Graduate School of Agricultural Sciences, Kagoshima University, Kagoshima, Japan

b Department of Food Life Sciences, Faculty of Fisheries, Kagoshima University, Kagoshima, Japan

c Kampo Research Laboratories, Kracie Pharma, Ltd., Toyama, Japan

d Pharmacological Department of Herbal Medicine, Graduate School of Medical and Dental Sciences, Kagoshima University, Kagoshima, Japan

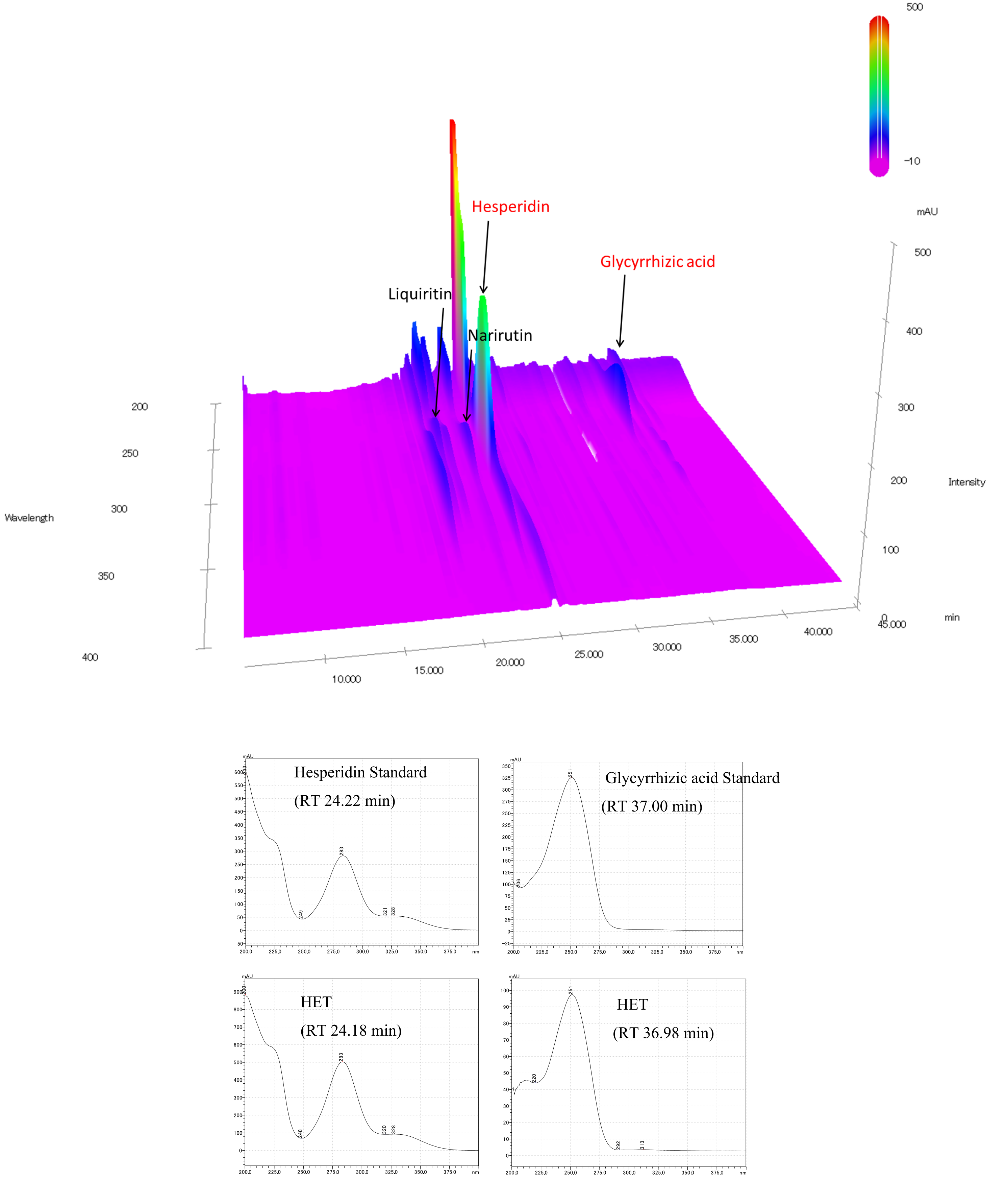

Supplementary Figure 1

3D-HPLC profile of HET. Each chemical marker in the HPLC profile was identified by comparison with retention times (RT) and UV spectra (200–400 nm) of their reference standards.

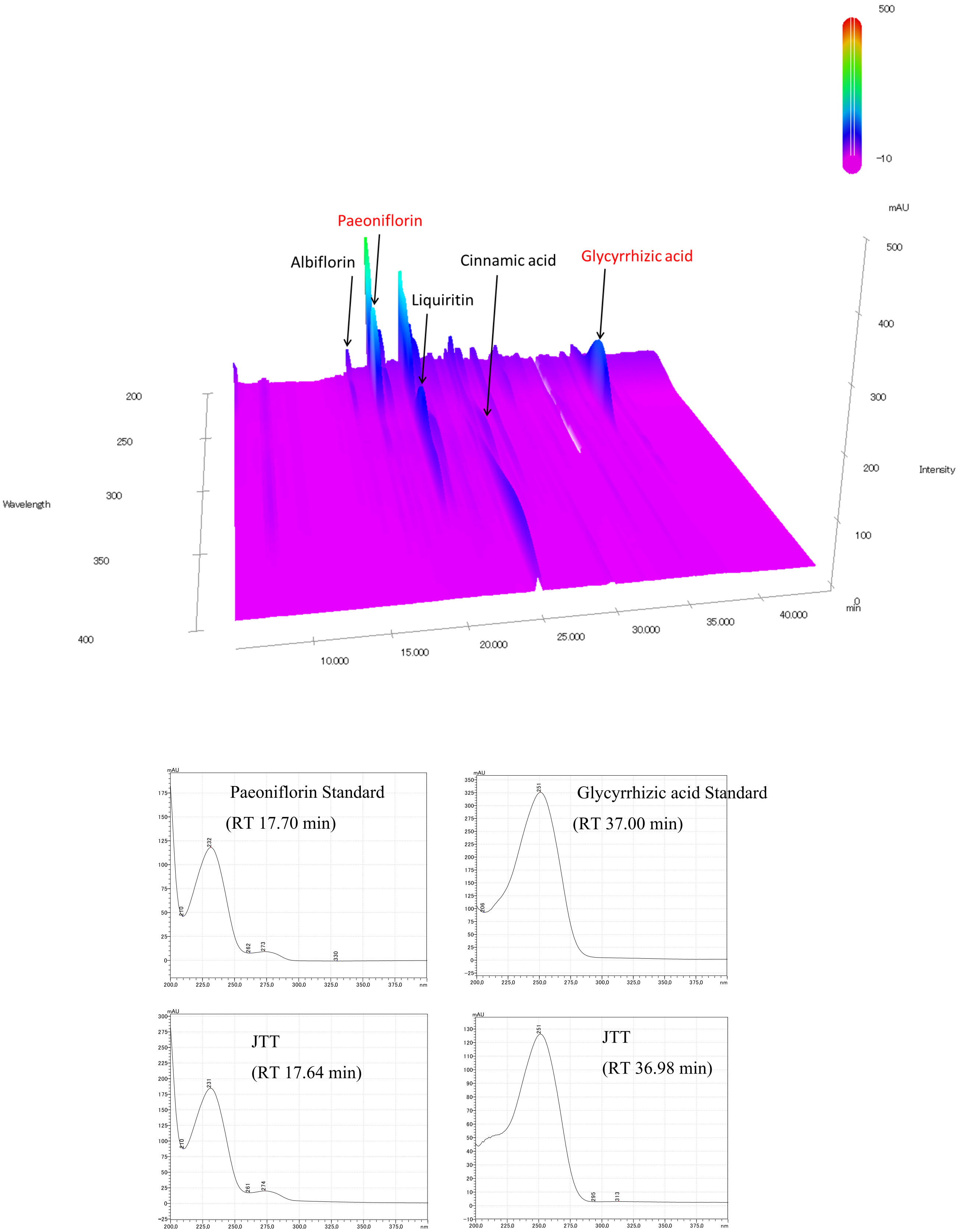

Supplementary Figure 2

3D-HPLC profile of JTT. Each chemical marker in the HPLC profile was identified by comparison with retention times (RT) and UV spectra (200–400 nm) of their reference standards.

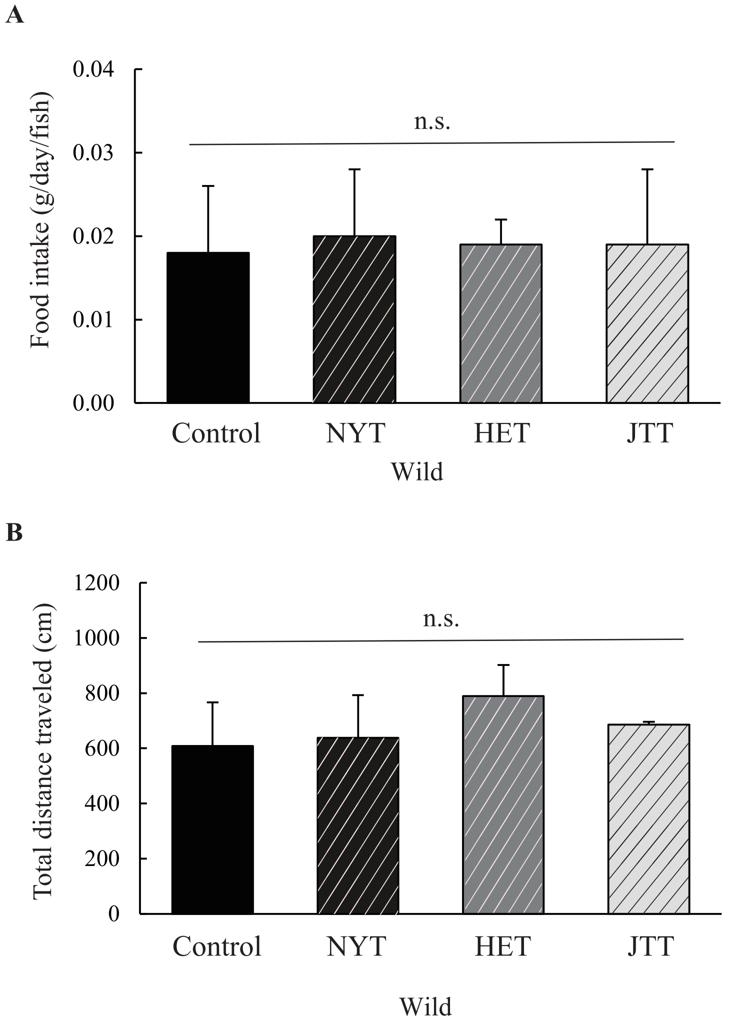

Supplementary Figure 3

Wild zebrafish were fed with control, NYT, HET, or JTT twice daily for four days (3% concentration of NYT, HET, and JTT in diet). Controls were fish fed feed without Kampo-hozai. Swimming mobility was estimated by the total distance traveled. (A) Food intake. n=6. (B) Total distance traveled. n=4. Results are shown as mean ± standard deviation. n.s., not significant..
